# Methylation in HT22 cells and primary hippocampal neurons with and without isoflurane exposurewhether isoflurane causes

**DOI:** 10.1186/s12871-020-00981-4

**Published:** 2020-03-14

**Authors:** Stefanie Klenke, Christian Specking, Maike Stegen, Andrea Engler, Jürgen Peters

**Affiliations:** Klinik für Anästhesiologie & Intensivmedizin, Universität Duisburg-Essen and Universitätsklinikum Essen, Hufelandstr. 55, D-45122 Essen, Germany

**Keywords:** Anesthetics, Volatile; Isoflurane, Cxcl12, Inflammation, Epigenetic changes, Gene expression, Methylation

## Abstract

**Background:**

Epigenetic modulation may play a role in anesthesia related phenotypes, such as cognitive impairment or memory loss, especially with exposure to anesthetics in the vulnerable phase of brain development. While isoflurane anesthesia can evoke neuroinflammation and neuroapoptosis in young animals, we investigated in a permanent hippocampal cell line (HT22) and in primary hippocampal neurons in an a priori in vitro analysis, whether isoflurane exposure 1) evokes DNA methylation changes in genes involved in apoptosis and inflammation, and 2) results observed in a permanent hippocampal cell line are comparable to primary hippocampal neurons. In case of methylation changes in specific genes, (3) mRNA analysis was performed to assess possible effects on gene expression.

**Methods:**

HT22 cells and primary mouse hippocampal neurons were exposed to 3% isoflurane for 4 h and DNA (each 6 single experiments) and RNA (3 single independent experiments) were extracted. Methylation analysis (EpiTect Methyl II PCR Array Systems, Qiagen) included the methylation status of 66 genes involved in apoptosis, cytokine production, inflammatory response, and autoimmunity. Quantitative Real-Time PCR was performed using the Quantitect SYBR Green Kit on a Step One Plus.

**Results:**

Methylation status was markedly different between immortalized HT22 cells and cultured primary hippocampal neurons without isoflurane exposure. Of 66 genes investigated, 29 were methylated to a significantly greater degree in HT22 cells compared to primary hippocampal neurons. In cultured primary hippocampal neurons, in contrast, there was a greater methylation in several genes involved in inflammation, accompanied with significant downregulation of C-X-C motif chemokine 12 with isoflurane exposure (*p* = 0.023).

**Conclusions:**

We demonstrate marked differences in gene methylation between HT22 cells and cultured primary hippocampal neurons without isoflurane exposure, with a greater methylation of several genes involved in inflammation upon isoflurane exposure and significant downregulation of *Cxcl12* mRNA expression in primary hippocampal neurons. Accordingly, further investigations of anesthesia related DNA methylation should be performed with special consideration being given to the choice of cells targeted for such investigations.

## Background

Environmental influences are translated by epigenetic mechanisms into expression changes of target genes, which may have significant roles in brain development. Administration of anesthetics also represents an environmental influence which can induce epigenetic changes, especially when exposed in the vulnerable phase of brain development. As an example, administration of ethanol, one of the oldest anesthetics known, during critical stages of brain development, results in significant chromatin remodelling [1, 2] in the promoters of several genes leading to long-term cognitive impairments, such as the brain-derived neurotrophic factor (BDNF) and c-Fos genes [1, 3]. Furthermore, general anesthesia can induce histone modification, resulting in suppression of, e.g., mouse Per2 expression in the suprachiasmatic nucleus [4] and c-Fos and BDNF histones within the immature rat hippocampus [5]. A further study revealed increased occupancy and methylation levels at the promoter region of the BDNF accompanied by suppressed BDNF expression and subsequent memory loss [6].

Together, these studies provide hints that epigenetic modulation may play a role in anesthesia related phenotypes. While isoflurane anesthesia can evoke neuroinflammation [7] and neuroapoptosis [8–10] in animal experiments, we investigated in this a priori in vitro analysis, whether isoflurane causes DNA methylation changes in an array of genes involved in apoptosis, cytokine production, and inflammatory response. We also assessed whether results obtained in the permanent hippocampal cell line HT22 are comparable with those in primary cultured hippocampal neurons. If results were comparable, further research, of cause, would be much easier than obtaining and maintaining in culture postnatal primary hippocampal neurons.

Therefore, we measured and compared methylation of an array of genes of HT22 cells and primary hippocampal neurons without and after isoflurane exposure. In case of changes in methylation of specific genes, mRNA analysis was performed to assess possible effects on gene expression.

## Methods

### Cell lines

HT22 is an immortalized mouse hippocampal cell line, which is a sub-line derived from parent HT4 cells that were originally immortalized from cultures of primary mouse hippocampal neurons [1]. The HT22 cells were a generous gift from the Salk Institute (La Jolla, CA, USA). HT22 cells were routinely maintained in Dulbecco’s modified Eagle’s medium (DMEM) supplemented with 10% fetal bovine serum, and in a 5% CO2 atmosphere at 37 °C.

Primary mouse hippocampal neurons were purchased from Lonza (M-HI-401, LOT numbers: 030807, 301,006, and 240,807, Basel, Switzerland). Mouse hippocampal neurons were isolated from E16–17, CD-1 mice which had been killed by decapitation, and then cryopreserved at primary passage. They stain positive for PGP and Tuj-1 and test negative for mycoplasma. Preparation of medium, coating of cell culture plates, thawing of cells / initiation of culture process, and maintenance of the cell culture was performed according to the manufacturer’s instructions. On day 1 of each experiment, thawing of cells and initiation of the culture process was started. Cells were seeded for experiments on day 4, 5, or 6 and underwent isoflurane or control exposures on the following day (day 5,6, or 7). The morphology of cultured cells was assessed once daily by microscopy.

### Cell exposures

For isoflurane exposure, HT22 cells or primary hippocampal neurons were transferred into a Billups-Rothenburg chamber (MIC-101, Billups-Rothenburg, Del Mar, CA) [11]. An in-line calibrated anesthetic agent Vaporizer (Dräger, Lübeck, Germany) was used to deliver isoflurane to the gas phase of the culture wells. Control air (5% CO2, 21% O2, balance nitrogen) (Air Liquide, Düsseldorf, Germany) and 3% isoflurane were delivered into the chamber with 5 l/min over 20 min. Effluent isoflurane, and carbon dioxide concentrations were monitored during flushing the chamber via a sampling port connected to an anesthetic agent analyzer (DrägerVamos, Lübeck, Germany). The chamber was then placed immediately into an incubator and remained there for 4 h. Isoflurane concentrations (3%) were again checked after 30, 90, and 180 min after exposure while flushing the chamber for 5 min. Control experiments were performed in the same manner, except that no isoflurane was added.

### Experimental protocol

Three hundred thousand HT22 cells were seeded in 6-well plates and underwent isoflurane exposure or control experiments without isoflurane. The experiments were repeated 6 times.

Primary hippocampal neurons were cultivated according to the manufacturer’s instructions. On culture days 4, 5, and 6 200.000 cells were seeded on a 24-well plate and underwent isoflurane or control exposures the following day (day 5, 6 or 7). For methylation analysis, this experiment was performed twice, resulting in 6 single experiments. Therefore, in the methylation analysis of primary hippocampal neurons, 2 experiments performed on day 5, 2 experiments performed on day 6, and 2 experiments performed on day 7 were included. For analysis of mRNA expression in primary hippocampal neurons, 3 independent experiments, in which cells underwent isoflurane or control exposures at day 5, 6 or 7 were included.

Immediately after 4 h of isoflurane exposure, DNA and RNA were extracted from the respective cells.

### Methylation analysis

DNA was extracted with the DNeasy Blood & Tissue Kits (Qiagen, Hilden, Germany) and DNA concentration was measured by a spectrometer (Eppendorf, Hamburg, Germany).

Methylation of genes was analyzed with EpiTect Methyl II PCR Array Systems (Qiagen, Hilden, Germany), which examines the methylation status across a CpG-rich sequence in gene promoters. The method is based on the detection of remaining input DNA after cleavage with a methylation-sensitive and/or a methylation-dependent restriction enzyme (EpiTect Methyl II DNA Restriction Kit, Qiagen, Hilden, Germany). These enzymes digest unmethylated and methylated DNA, respectively. Following digestion, the remaining DNA is quantified by real-time PCR (RT^2^ SYBR® Green qPCR Mastermix, Qiagen, Hilden, Germany) in each individual enzyme reaction using primers that flank a promoter (gene) region of interest. We analyzed the DNA methylation status of 22 genes of the pathways focused EpiTect Methyl II PCR Array Systems (Qiagen, Hilden, Germany), i.e., apoptosis (EAMM-121Z, Table 1), cytokine production (EAMM-541Z, Table 2), and inflammatory response and autoimmunity (EAMM-521Z, Table 3). The relative fractions of methylated and unmethylated DNA are subsequently determined by comparing the amount in each digest with that of a mock (no enzymes added) digest using the ΔCt method as recommended by the manufactor (Qiagen, Hilden, Germany).

**Table 1:** Apoptosis Array

Gene	Description	Gene ID	Function
Apaf1	apoptotic peptidase activating factor 1	11,783	Caspase & Regulator
Bad	BCL2-associated agonist of cell death	12,015	Induction of Apoptosis Positive Regulator of Apoptosis
Bax	BCL2-associated X protein	12,028	Induction of Apoptosis Anti-Apoptosis Positive Regulator of Apoptosis Caspase & Regulator
Bcl2l11	BCL2-like 11	12,125	Induction of Apoptosis Positive Regulator of Apoptosis
Bclaf1	BCL2-associated transcription factor 1	72,567	Induction of Apoptosis Positive Regulator of Apoptosis
Bid	BH3 interacting domain death agonist	12,122	Induction of Apoptosis Positive Regulator of Apoptosis
Bik	BCL2-interacting killer	12,124	Induction of Apoptosis Positive Regulator of Apoptosis
Birc2	baculoviral IAP repeat-containing 2	11,797	Negative Regulator of Apoptosis
Bnip3l	BCL2/adenovirus E1B interacting protein 3-like	12,177	Induction of Apoptosis Anti-Apoptosis Negative Regulator of Apoptosis Positive Regulator of Apoptosis
Casp3	caspase 3	12,367	Induction of Apoptosis Negative Regulator of Apoptosis Caspase & Regulator
Casp9	caspase 9	12,371	Caspase & Regulator
Cideb	cell death-inducing DNA fragmentation factor, alpha subunit-like effector B	12,684	Induction of Apoptosis Positive Regulator of Apoptosis
Cradd	CASP2 and RIPK1 domain containing adaptor with death domain	12,905	Induction of Apoptosis Positive Regulator of Apoptosis Caspase & Regulator
Dapk1	death associated protein kinase 1	69,635	Induction of Apoptosis Anti-Apoptosis Negative Regulator of Apoptosis
Dffa	DNA fragmentation factor, alpha subunit	13,347	Induction of Apoptosis Negative Regulator of Apoptosis
Fadd	Fas (TNFRSF6)-associated via death domain	14,082	Induction of Apoptosis Positive Regulator of Apoptosis
Gadd45a	growth arrest and DNA-damage-inducible 45 alpha	13,197	Induction of Apoptosis
Hrk	harakiri, BCL2 interacting protein	12,123	Induction of Apoptosis Anti-Apoptosis Positive Regulator of Apoptosis
Ltbr	lymphotoxin B receptor	17,000	Induction of Apoptosis
Tnfrsf21	tumor necrosis factor receptor superfamily, member 21	94,185	Induction of Apoptosis
Tnfrsf25 (Dr3)	tumor necrosis factor receptor superfamily, member 25	85,030	Induction of Apoptosis Positive Regulator of Apoptosis
Trp53	transformation related protein 53	22,059	Induction of Apoptosis Negative Regulator of Apoptosis Positive Regulator of Apoptosis Caspase & Regulator

**Table 2:** Cytokine Array

Gene	Description	Gene ID	Function
Bcl10	B cell leukemia/lymphoma 10	12,042	T & B Cell Function Regulator Transcriptional Regulator Cytokine Production Signaling Molecule
Bcl3	B cell leukemia/lymphoma 3	12,051	T & B Cell Function Regulator Transcriptional & Translational Regulator Cytokine Production Signaling Molecule
Cd14	CD14 antigen	12,475	Environment & Intracellular Stimuli Response
Furin	furin	18,550	T Cell Function Regulator
Gata3	GATA binding protein 3	14,462	Transcriptional Regulator Environment & Intracellular Stimuli Response
Hmox1	heme oxygenase 1	15,368	T Cell Function Regulator Environment & Intracellular Stimuli Response Cytokine Production Signaling Molecule
Igf2bp2	insulin-like growth factor 2 mRNA binding protein 2	319,765	Translational Regulator
Il18	interleukin 18	16,173	T Cell Function Regulator
Il9r	interleukin 9 receptor	16,199	Environment & Intracellular Stimuli Response
Inha	inhibin alpha	16,322	B Cell Function Regulator
Irf1	interferon regulatory factor 1	16,362	Transcriptional Regulator
Ltb	lymphotoxin B	16,994	Environment & Intracellular Stimuli Response Cytokine Production Signaling Molecule
Malt1	MALT1 paracaspase	24,354	T Cell Function Regulator Environment & Intracellular Stimuli Response Cytokine Production Signaling Molecule
Map 3 k7	mitogen-activated protein kinase kinase kinase 7	26,409	T Cell Function Regulator Cytokine Production Signaling Molecule
Myd88	myeloid differentiation primary response gene 88	17,874	Environment & Intracellular Stimuli Response Cytokine Production Signaling Molecule
Prkca	protein kinase C, alpha	18,750	Environment & Intracellular Stimuli Responses
Smad3	SMAD family member 3	17,127	Transcriptional Regulator Environment & Intracellular Stimuli Response Cytokine Production Signaling Molecule
Smad4	SMAD family member 4	17,128	Transcriptional Regulator
Sod1	superoxide dismutase 1, soluble	20,655	T Cell Function Regulator Environment & Intracellular Stimuli Response Cytokine Production Signaling Molecule
Stat5a	signal transducer and activator of transcription 5A	20,850	T & B Cell Function Regulator Transcriptional Regulator Environment & Intracellular Stimuli Response Cytokine Production Signaling Molecule
Traf2	TNF receptor-associated factor 2	22,030	T Cell Function Regulator Cytokine Production Signaling Molecule
Traf6	TNF receptor-associated factor 6	22,034	T Cell Function Regulator Cytokine Production Signaling Molecule

**Table 3:** Inflammatory Response & Autoimmunity Array

Gene	Description	Gene ID	Function
Aft2	activating transcription factor 2	11,909	Inflammatory Response & Autoimmunity Gene
Cx3cl1	chemokine (C-X3-C motif) ligand 1	20,312	Cytokine Production Cytokine
Cxcl12	chemokine (C-X-C motif) ligand 12	20,315	Cytokine
Cxcl14	chemokine (C-X-C motif) ligand 14	57,266	Cytokine
Fadd	Fas (TNFRSF6)-associated via death domain	14,081	Inflammatory Response & Autoimmunity Gene
Gata3	GATA binding protein 3	14,462	Inflammatory Response & Autoimmunity Gene
Il10ra	interleukin 10 receptor, alpha	16,154	Cytokine Receptor
Il11	interleukin 11	16,156	Cytokine
Il13	interleukin 13	16,163	Cytokine
Il13ra1	interleukin 13 receptor, alpha 1	16,164	Cytokine Receptor
Il17ra	interleukin 17 receptor A	16,172	Cytokine Receptor
Il18	interleukin 18	16,173	Cytokine
Il4ra	interleukin 4 receptor, alpha	16,190	Cytokine Receptor
Il6ra	interleukin 6 receptor, alpha	16,194	Cytokine Production Cytokine Receptor
Il6st	interleukin 6 signal transducer	16,195	Cytokine Receptor
Il7	interleukin 7	16,196	Cytokine
Inha	inhibin alpha	16,322	Inflammatory Response & Autoimmunity Gene
Ltb	lymphotoxin B	16,994	Cytokine Production Cytokine
Mif	macrophage migration inhibitory factor	17,319	Cytokine
Tbck	TBC1 domain containing kinase	271,981	Inflammatory Response & Autoimmunity Gene
Tgfb1	transforming growth factor, beta 1	21,803	Inflammatory Response & Autoimmunity Gene
Tyk2	tyrosine kinase 2	54,721	Inflammatory Response & Autoimmunity Gene

### Quantitative real-time PCR

RNA was extracted with peqGOLD MircoSpin Total RNA Kits (Peqlab Biotechnology, Erlangen, Germany) at the indicated time point. RNA concentration was measured by a spectrometer (Eppendorf, Hamburg, Germany). First-strand cDNA was synthesized from 1 μg of total RNA with oligo-dT primers (Roche, Mannheim, Germany) by using Superscript II reverse transcriptase, as recommended by the supplier (Invitrogen, Carlsbad, CA). Relative quantification of mRNA expression was carried out by using primers for those genes whose methylation level showed a difference greater than 20% among in the primary hippocampal neurons with and without isoflurane exposure (*Cxcl12, Cxcl14, Gata3, Il11, Il13, and Il4ra,*; for primer details see supplementary Table 1, for gene details see Table 3), taking several considerations of reference gene selection into account [11]. Contamination with genomic DNA was circumvented by DNase treatment of RNA and by using RNA as template. The PCR reaction mix was prepared using the Quantitect SYBR Green Kit (Qiagen, Hilden, Germany) following the manufacturer’s instructions. PCR was performed in the Step One Plus (Applied Biosystems, Foster City, USA). Real-Time PCR experiments were performed three times.

### Statistical analysis

Data are presented as means ± standard deviation (SD). Data were analyzed using Graph Pad Prism 6 (Graph Pad Software, La Jolla, CA). Methylation analysis was done according to the manufacturer’s instructions. A change in methylation of > 20% was considered as significant, according to the instructions of the manufacturer and comparable to other studies using the EpiTect Methyl II PCR Array System [12, 13]. Here, DNA methylation was compared between apoptosis-related genes in pleomorphic adenomas of the salivary glands [13], respectively in genes involved in inflammation and autoimmunity in inflammatory bowel disease [12]. mRNA expression was done in genes with significant methylation changes, according to references [12, 13], and analyzed with an unpaired t-test. This An a priori alpha error p of less than 0.05 was considered to indicate statistical significance.

## Results

### Methylation of HT22 cells and primary hippocampal cells without isoflurane exposure

**Fig. 1:**
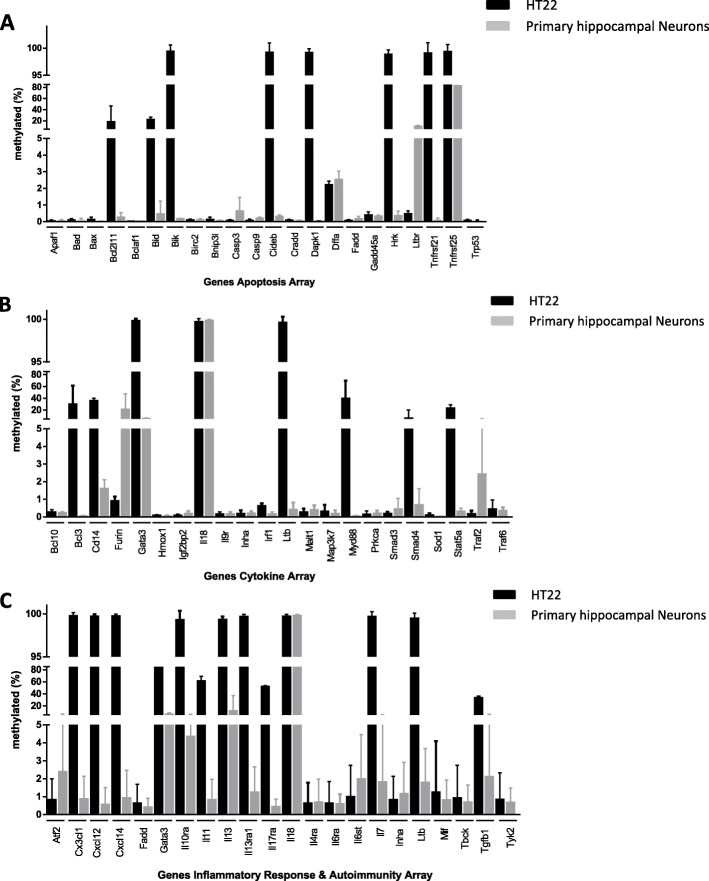
Methylation of genes in the functional gene panels of HT22 cells (black columns) and primary cultured hippocampal neurons (grey columns). Data represent means ± standard deviation (SD) and were obtained from 6 single experiments. Gene details are given in Tables 1, 2, and 3. **a** Apoptosis gene panel. **b** Cytokine gene panel. **c** Inflammatory gene panel

In a first step, we measured methylation of genes in HT22 and in primary hippocampal neurons in control experiments without exposure to isoflurane (Fig. 1a-c). Interestingly, the methylation status between the immortalized cell line HT22 and the primary hippocampal neuron cultures was markedly different. In general, methylation of genes in HT22 cells was greater than in the primary cells. Eight of 22 genes of the apoptosis array (*Bid, Bik, Cideb, Dapk1, Hrk, Ltbr, Tnfrsf21*, and *Tnfrsf25*) were significantly greater (> 20%) methylated in HT22 cells compared to primary hippocampal neurons (Fig. 1a). For example, methylation of *Bik* was 99.4% in HT22 cells, whereas it was 0.2% in primary hippocampal neurons (Fig. 1A). In the cytokine array, genes *Bcl10, Bcl3*, *Cd114, Furin, Gata3, Irf1, Ltb*, *Myd88*, and *Stat5a* showed > 20% methylation changes in the HT22 cells compared to the primary hippocampal neurons (e.g. *Ltb*: 99.6% vs. 0.4%) (Fig. 1b). Most differences were found in genes of the inflammatory array. Here, 12 of 22 genes (*Cx3cl1, Cxcl12, Cxcl14, Gata3, Il10ra, Il11, Il13, Il13ra1, Il17ra, Il7, Ltb, and Tgfb1)* were significantly greater (> 20%) methylated in HT22 cells compared to primary hippocampal neurons (Fig. 1c). Methylation of *Cxcl12* was 99.7% in HT22 cells, whereas it was 0.6% in primary hippocampal neurons (Fig. 1c). Only one gene (*Il18*) was nearly completely methylated in the primary neurons (> 99.8%).

### Influence on isoflurane exposure on methylation in primary hippocampal cells

**Fig. 2:**
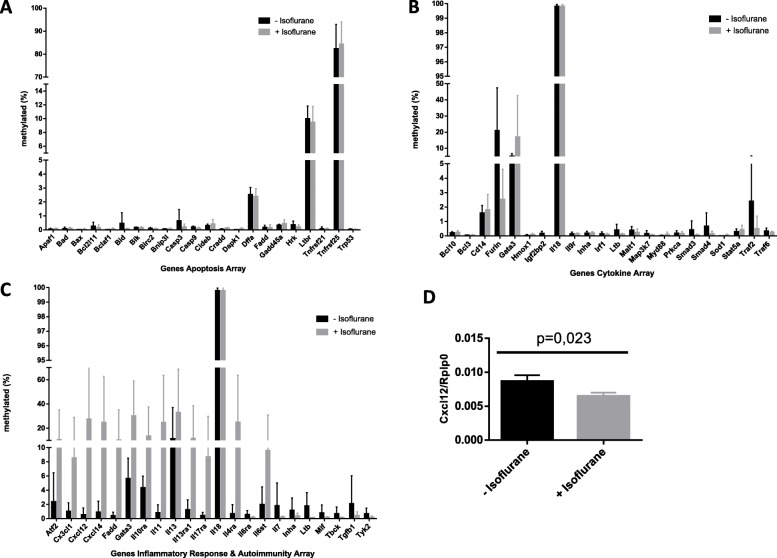
**a-c** Methylation of genes in various functional gene panels in primary hippocampal neurons without isoflurane exposure (black columns) and with isoflurane exposure (grey columns). Data represent means ± standard deviation (SD) and were obtained from 6 single experiments. Gene details are given in Tables 1, 2, and 3. **a** Apoptosis gene panel. **b** Cytokine gene panel. **c** Inflammatory gene panel. **d***Cxcl12* mRNA expression analysis in primary hippocampal neurons with and without exposure to isoflurane. Data are mean ± standard deviation (SD), obtained from 3 single experiments, and analyzed by students unpaired t-test.

We measured whether isoflurane exposure alters methylation of genes in primary hippocampal neurons (Fig. 2). However, no changes were seen for genes of the apoptosis array (Fig. 2a) and the cytokine array (Fig. 2b). There was a greater than 20% methylation change for several genes in the inflammatory array after isoflurane exposure: *Cxcl12, Cxcl14, Gata3, Il11, Il13, and Il4ra* (Fig. 2c). Methylation of *Cxcl12* was 0.6% without, but 27.5% with isoflurane exposure. For all genes, variation between the single experiments was seen (Fig. 2c). In HT22 cells, neither methylation of the genes in the apoptosis array (A), nor those in the cytokine (B) or in the inflammatory arrays (C) was altered by isoflurane (Supplementary Fig. 1).

### mRNA expression analysis of inflammatory genes in primary cells

To elucidate whether greater methylation of several genes in the inflammatory array with isoflurane exposure altered mRNA expression of these genes, RT primer were designed for genes, in which methylation was > 20% different after isoflurane exposure (*Cxcl12, Cxcl14, Gata3, Il11, Il13, and Il4ra*, for primer sequences see supplementary Table 1). mRNA expression was then measured in cDNA samples of cells exposed to isoflurane and in cells under control conditions. Expression of *Cxcl12* was significantly lower under isoflurane exposure (*p* = 0.023, Fig. 2d), whereas no differences were found for the genes *Cxcl14,* and *Gata3 (*data not shown). No RT product could be obtained for genes *Il11, Il13,* and *Il14ra.*

## Discussion

In our study, we demonstrate wide differences in the gene methylation status between the HT22 cell line and primary hippocampal neurons in the absence of isoflurane exposure, but greater methylation of several genes involved in inflammation upon isoflurane exposure and significant downregulation of *Cxcl12* mRNA expression in primary hippocampal neurons. This could represent further epigenetic mechanisms of anesthesia related phenotype in the immature brain. Our results suggest that further studies investigating epigenetic effects of isoflurane or other volatile anesthetics should not be performed on HT22 cells but on primary hippocampal neurons, albeit technically more difficult.

A greater level of methylation was seen in the cell line HT22 than in primary hippocampal neurons. There are only few data on DNA methylation in immortalized cell lines compared to respective primary cells [14–16]. In general, there are cell-type specific methylation signatures preserved in primary cell lines and tissues as well as methylation differences induced by cell culture [16]. For example, the Forkhead box protein L2 promoter is hypermethylated in immortalized but not in primary cells [14]. This argues against a use of HT22 cells to study epigenetic changes, e.g., induced by volatile anesthetics.

In the primary hippocampal neurons, no differences were seen in genes in pathways of cytokine production and apoptosis, but there was a greater methylation of several genes in inflammatory pathways.

Isoflurane anesthesia can evoke neuroinflammation [7] and methylation of genes in the inflammatory pathway may be one molecular mechanism leading to neuroinflammation. Epigenetic mechanisms for isoflurane induced neuroinflammation have already been reported for the aged brain: isoflurane exposure led to declines in chromatin histone acetylation with resulting downregulation of the BDNF- tyrosine kinase receptor B signaling pathway and with increased levels of inflammatory Interleukin 1 beta and Interleukin 6 in aged rats [17]. Our study, therefore, may first indicate that epigenetic modulation of inflammatory genes may also play a role in the volatile anesthetic induced neuroinflammation in the immature brain and this should be investigated in further studies.

Special regard may be given to the C-X-C motif chemokine 12 (CXCL12), whose mRNA was significantly downregulated, while no differences were found for the genes *Cxcl14* and *Gata3* and no Real-Time PCR product could be obtained for genes *Il11, Il13,* and *Il14ra.* It might be speculated that expression of these genes is too low to become detective.

CXCL12 is a 68-amino-acid CXC chemokine with an essential role in several important processes such as vascular and neuronal development and hematopoiesis [18]. The CXCL12 response takes place at a very early stage of embryonic development and seems to be broadly operative whenever cell migration is required [19]. Mice lacking CXCL12 die prenatally and exhibit defects in vascularization, neuronal development, and hematopoiesis [20]. CXCL12 could be a key chemokine regulating the entrance of lymphocytes into the CNS [21] and immune factors, such as TNFα and interleukins might influence CXCL12 expression [22–25]. In further studies, a promoter characterization of the genes of interest, such as the *Cxcl12* promoter, with special regard to the CpG islands, would be of great importance. The EpiTect Methyl II PCR Array System examines the methylation status across a CpG-rich sequence in gene promoter, however, the exact length of the amplified CpG island/promoter and the physical location of the annealing sites of the PCR primers are not disclosed.

Limitations of the study should be mentioned. One important item is the relevant dose and duration of isoflurane administration necessary to evoke DNA methylation changes, if any, and previous data are missing. Previous animal studies are difficult to interpret since long durations and supraanesthetic doses of isoflurane were administered to young animals [26]. While such intense exposures are maybe needed to investigate possible neuropathological and other pathological changes, these conditions hardly relate to human clinical practice. We chose an isoflurane concentration of 3 vol% inspired but a MAC value cannot be obtained for cell lines. Since the primary hippocampal neurons were exposed to isoflurane on culture days 5, 6, and 7 (mouse hippocampal neurons were isolated from E16–17 mice), therefore, one might speculate that the 3% isoflurane corresponds to 1 MAC [27], which is a usual clinical dose.

To this end, it also needs to be questioned whether the observed effect of isoflurane is transferable to other volatile anesthetics such as sevoflurane. In a landmark paper, neuroapoptotic properties of equipotent anesthetic concentrations of desflurane, isoflurane, or sevoflurane were compared in neonatal mice and similar neurotoxic profiles were found [27]. However, whether this finding holds true only for only this condition or has to be tested as research hypothesis needs to be clarified.

Furthermore, it needs to be questioned, whether exposure of the primary hippocampal neurons on days 5, 6, and 7 was plausible. We believe that this time frame represents a vulnerable phase of brain development. Exposure on three following days was done due to practical and cost reasons, however, analysis of neurons on always the same day may result in different conclusions. Exposure of neurons on three following days may although partly explain the variation of methylation in the experiments, which was only seen in the primary hippocampal neurons. It is important to note, that RNA for mRNA expression analysis was obtained from three independent experiments, indicating that our experimental setup was reliable.

For interpreting the results of the EpiTect Methyl II PCR Array System, we considered a methylation change of > 20% as significant. According to the manufacturer, the significance of the level of methylated DNA must be defined by the researcher. Methylated DNA may have biological significance if such methylation status is associated with a specific phenotype and measuring the corresponding gene expression between samples is recommended. While we did not investigate phenotype changes, we observed significant differences in mRNA expression.

## Conclusions

In conclusion, we demonstrate wide differences in methylation status between HT22 cells and primary cultured hippocampal neurons, with increased methylation in HT22 cells, suggesting that further epigenetic investigations on effects of isoflurane should not be performed in HT22 cells. Furthermore, upon isoflurane exposure in hippocampal neurons we found a greater DNA methylation from several genes involved in inflammation with significant downregulation of *Cxcl12* mRNA expression. Based on this a priori analysis, further investigations addressing possible DNA methylation changes in inflammatory genes due to exposure to anesthetics should be performed.

Full name of all genes is given in Tables 1, 2, and 3.

## Supplementary information

**Additional file 1.**

**Additional file 2.**

## Data Availability

The datasets used and/or analyzed during the current study are available from the corresponding author on reasonable request.

## Abbreviations

BDNF: Brain-derived neurotrophic factor
DNA: Deoxyribonucleic acid
GABA: Gamma-aminobutyric acid
MAC: Minimum alveolar concentration
mRNA: Messenger RNA
NMDA: N-methyl-D-aspartate
RNA: Ribonucleic acid
