# Correction to: Methylation in HT22 cells and primary hippocampal neurons with and without isoflurane exposure

**DOI:** 10.1186/s12871-020-00989-w

**Published:** 2020-04-03

**Authors:** Stefanie Klenke, Christian Specking, Maike Stegen, Andrea Engler, Jürgen Peters

**Affiliations:** Klinik für Anästhesiologie & Intensivmedizin, Universität Duisburg-Essen and Universitätsklinikum Essen, Hufelandstr. 55, D-45122 Essen, Germany

**Correction to: BMC Anesthesiol (2020) 20:66**

**https://doi.org/10.1186/s12871-020-00981-4**

Following publication of the original article [1], it was brought to our attention of an error in the article title. The article title should read “Methylation in HT22 cells and primary hippocampal neurons with and without isoflurane exposure”.

The original article has been corrected.

The publisher apologizes for any inconvenience caused by this error.
