# Supplementary material for: Methylation in HT22 cells and primary hippocampal neurons with and without isoflurane exposurewhether isoflurane causes
Source: BMC Anesthesiol. 2020 Mar 14;20:66. doi: 10.1186/s12871-020-00981-4 (PMC7071644; doi:10.1186/s12871-020-00981-4)
Supplement: Supplementary file 1 — Additional file 1. [file 12871_2020_981_MOESM1_ESM.docx]

**Supplementary Table1**

| **Name** | **Seq: 5´-> 3´ (forward)** | **Seq: 5´-> 3´ (reverse)** | **Refseq ^a^** |
| --- | --- | --- | --- |
| Cxcl12 | TGCATCAGTGACGGTAAACCA | GTTGTTCTTCAGCCGTGCAA | NM_021704 |
| Cxcl14 | ACCACCAAGAGCATGTCCAG | GTAGACCCTGCGCTTCTCG | NM_019568 |
| Gata3 | CCTTGCTACTCAGGTGATCG | GAGGAATCCGAGTGTGACCA | NM_008091 |
| Il11 | CACACTCACAAACCTCCCCT | CAGGCGACAAACACAGTTCAT | NM_008350 |
| Il13 | AGACCAGACTCCCCTGTGCA | TGGGTCCTGTAGATGGCATTG | NM_008355 |
| Il4ra | TGAGGAGAAGCAGAGGGACC | CACAAAAGGTGCCTGCACAAG | NM_001008700 |
| Rplp0 | AGATGCAGCAGATCCGCAT | GTTCTTGCCCATCAGCACC | NM_007475 |

^a^ Genebank accession number of cDNA
