# Supplementary material for: Methylation in HT22 cells and primary hippocampal neurons with and without isoflurane exposurewhether isoflurane causes
Source: BMC Anesthesiol. 2020 Mar 14;20:66. doi: 10.1186/s12871-020-00981-4 (PMC7071644; doi:10.1186/s12871-020-00981-4)
Supplement: Supplementary file 2 — Additional file 2. [file 12871_2020_981_MOESM2_ESM.pptx]

## Slide 1
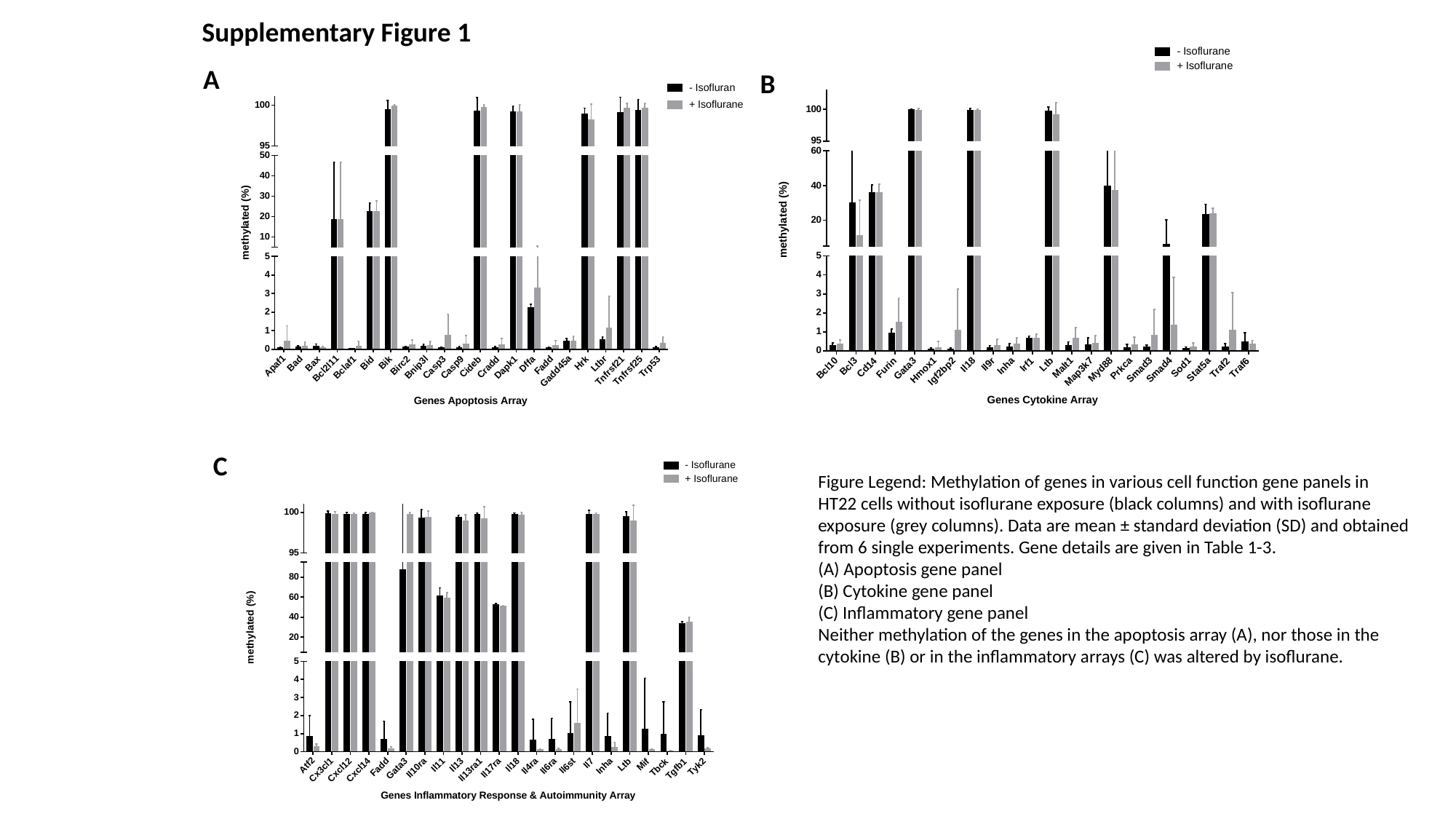

Supplementary Figure 1
A
B
C
Figure Legend: Methylation of genes in various cell function gene panels in HT22 cells without isoflurane exposure (black columns) and with isoflurane exposure (grey columns). Data are mean ± standard deviation (SD) and obtained from 6 single experiments. Gene details are given in Table 1-3.
(A) Apoptosis gene panel
(B) Cytokine gene panel
(C) Inflammatory gene panel
Neither methylation of the genes in the apoptosis array (A), nor those in the cytokine (B) or in the inflammatory arrays (C) was altered by isoflurane.
